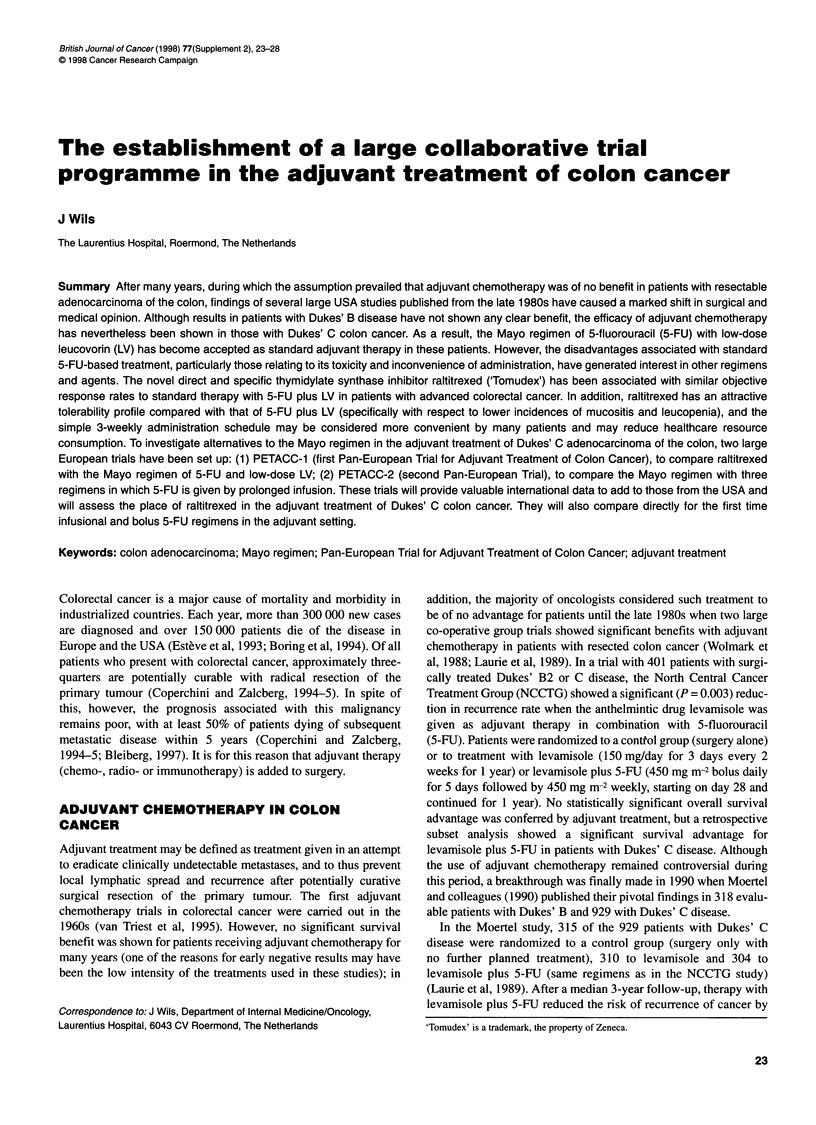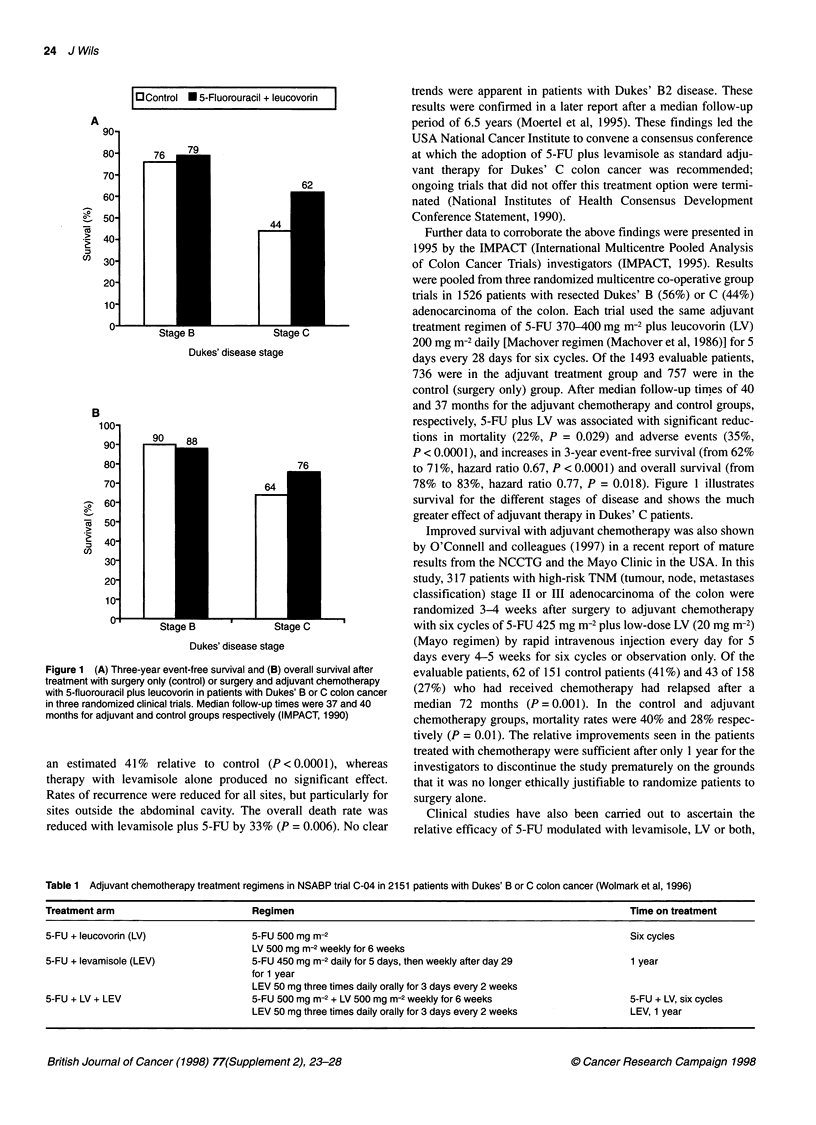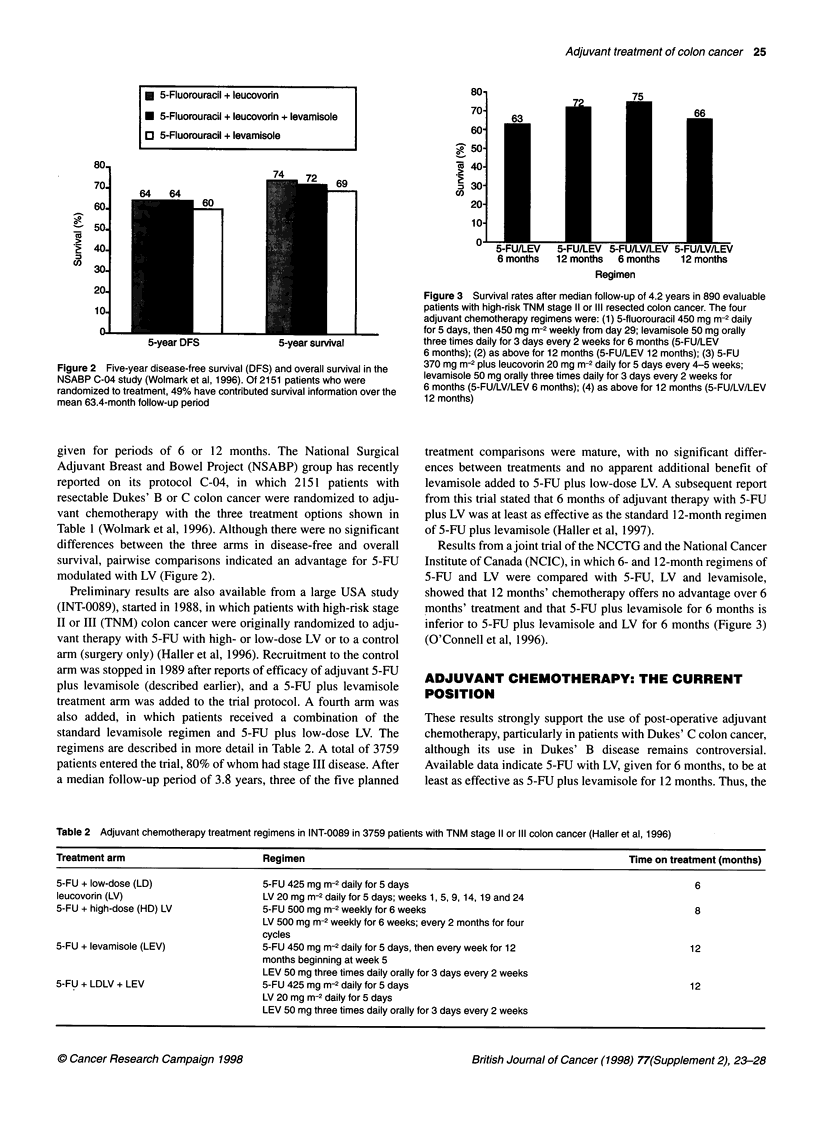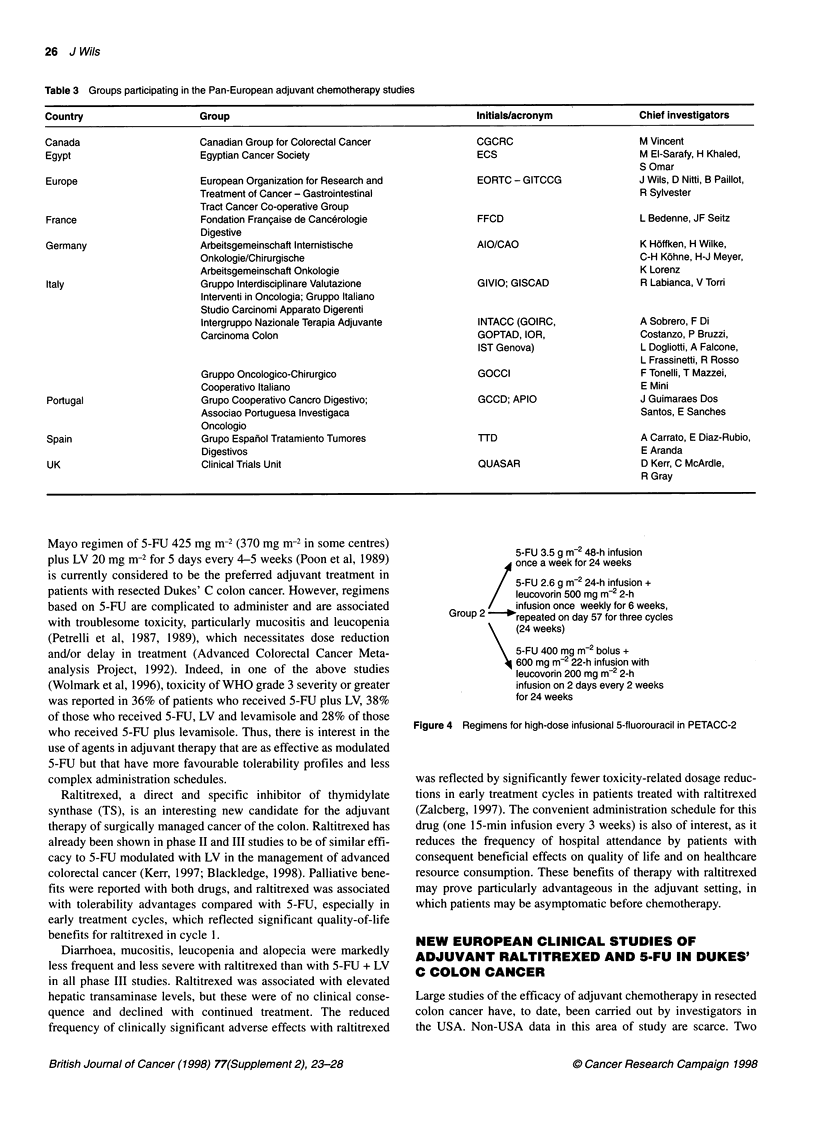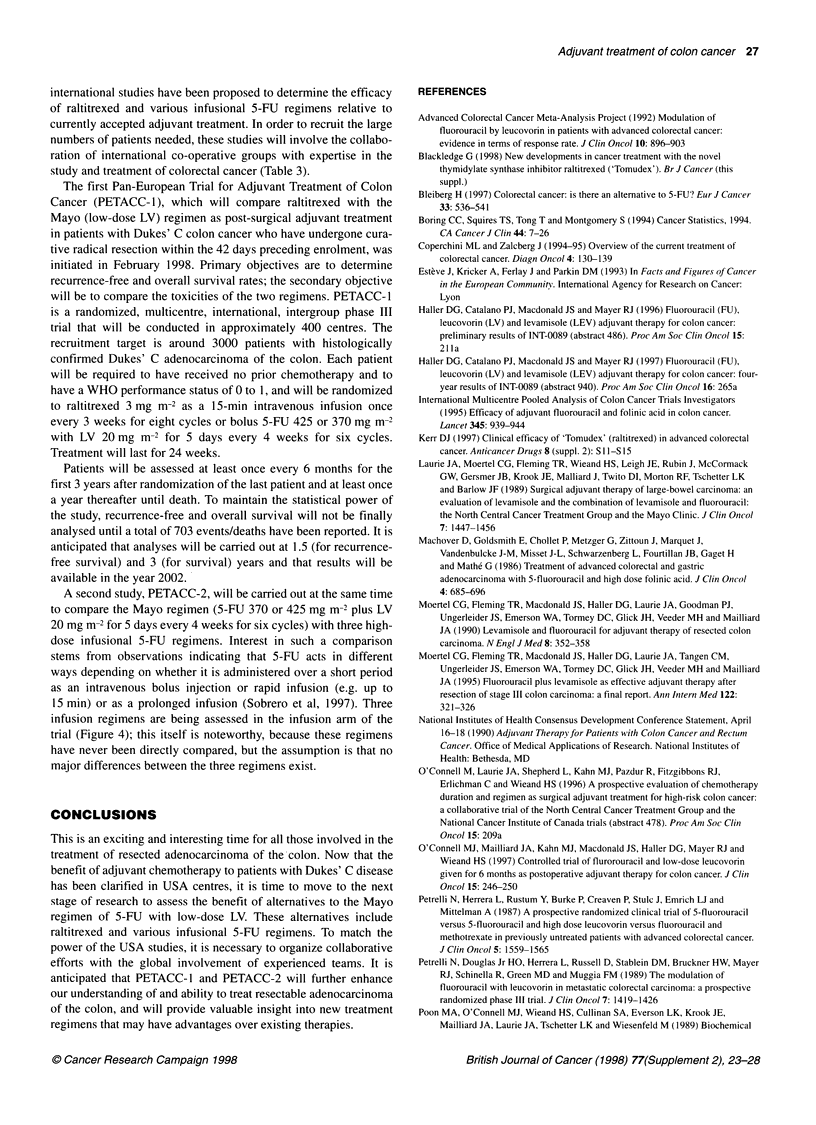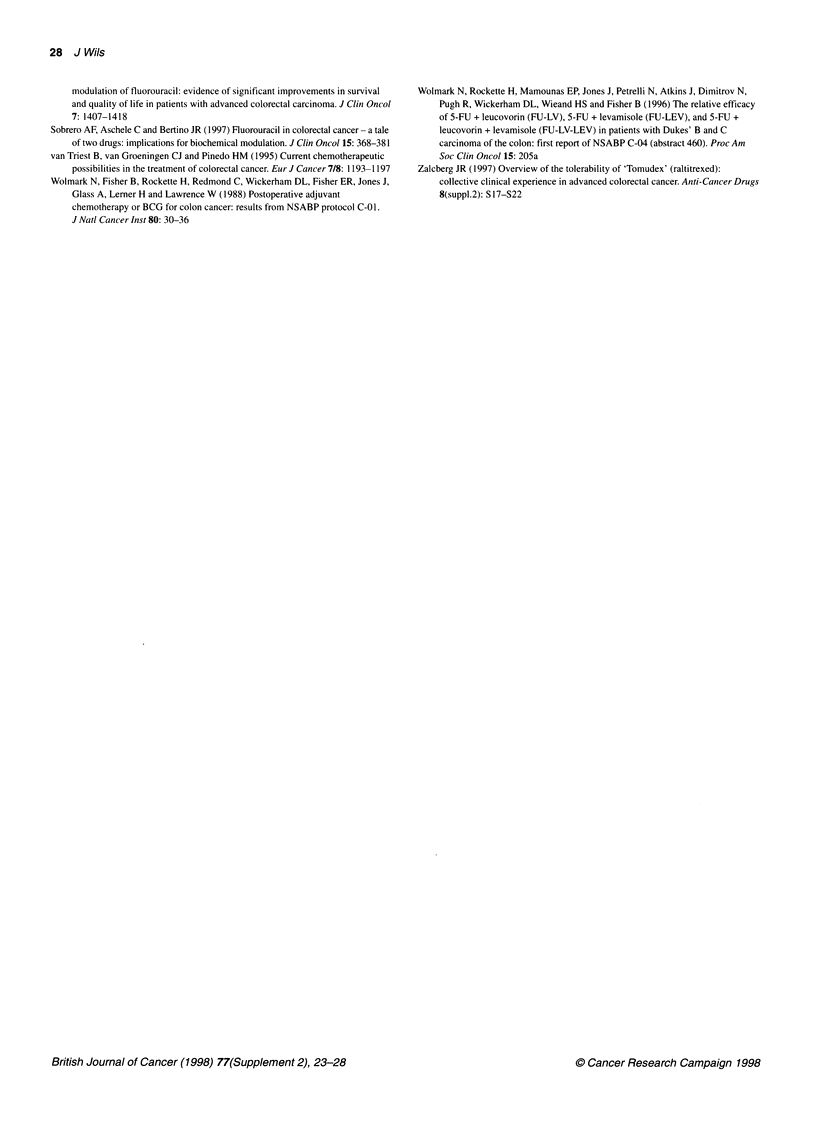# The establishment of a large collaborative trial programme in the adjuvant treatment of colon cancer.

**DOI:** 10.1038/bjc.1998.422

**Published:** 1998

**Authors:** J. Wils

**Affiliations:** The Laurentius Hospital, Roermond, The Netherlands.

## Abstract

After many years, during which the assumption prevailed that adjuvant chemotherapy was of no benefit in patients with resectable adenocarcinoma of the colon, findings of several large USA studies published from the late 1980s have caused a marked shift in surgical and medical opinion. Although results in patients with Dukes' B disease have not shown any clear benefit, the efficacy of adjuvant chemotherapy has nevertheless been shown in those with Dukes' C colon cancer. As a result, the Mayo regimen of 5-fluorouracil (5-FU) with low-dose leucovorin (LV) has become accepted as standard adjuvant therapy in these patients. However, the disadvantages associated with standard 5-FU-based treatment, particularly those relating to its toxicity and inconvenience of administration, have generated interest in other regimens and agents. The novel direct and specific thymidylate synthase inhibitor raltitrexed ('Tomudex') has been associated with similar objective response rates to standard therapy with 5-FU plus LV in patients with advanced colorectal cancer. In addition, raltitrexed has an attractive tolerability profile compared with that of 5-FU plus LV (specifically with respect to lower incidences of mucositis and leucopenia), and the simple 3-weekly administration schedule may be considered more convenient by many patients and may reduce healthcare resource consumption. To investigate alternatives to the Mayo regimen in the adjuvant treatment of Dukes' C adenocarcinoma of the colon, two large European trials have been set up: (1) PETACC-1 (first Pan-European Trial for Adjuvant Treatment of Colon Cancer), to compare raltitrexed with the Mayo regimen of 5-FU and low-dose LV; (2) PETACC-2 (second Pan-European Trial), to compare the Mayo regimen with three regimens in which 5-FU is given by prolonged infusion. These trials will provide valuable international data to add to those from the USA and will assess the place of raltitrexed in the adjuvant treatment of Dukes' C colon cancer. They will also compare directly for the first time infusional and bolus 5-FU regimens in the adjuvant setting.


British Journal of Cancer (1998) **77**(Supplement 2), 23–28
© 1998 Cancer Research Campaign

### The establishment of a large collaborative trial programme in the adjuvant treatment of colon cancer

**J Wils**

The Laurentius Hospital, Roermond, The Netherlands

**Summary** After many years, during which the assumption prevailed that adjuvant chemotherapy was of no benefit in patients with resectable adenocarcinoma of the colon, findings of several large USA studies published from the late 1980s have caused a marked shift in surgical and medical opinion. Although results in patients with Dukes' B disease have not shown any clear benefit, the efficacy of adjuvant chemotherapy has nevertheless been shown in those with Dukes' C colon cancer. As a result, the Mayo regimen of 5-fluorouracil (5-FU) with low-dose leucovorin (LV) has become accepted as standard adjuvant therapy in these patients. However, the disadvantages associated with standard 5-FU-based treatment, particularly those relating to its toxicity and inconvenience of administration, have generated interest in other regimens and agents. The novel direct and specific thymidylate synthase inhibitor raltitrexed ('Tomudex') has been associated with similar objective response rates to standard therapy with 5-FU plus LV in patients with advanced colorectal cancer. In addition, raltitrexed has an attractive tolerability profile compared with that of 5-FU plus LV (specifically with respect to lower incidences of mucositis and leucopenia), and the simple 3-weekly administration schedule may be considered more convenient by many patients and may reduce healthcare resource consumption. To investigate alternatives to the Mayo regimen in the adjuvant treatment of Dukes' C adenocarcinoma of the colon, two large European trials have been set up: (1) PETACC-1 (first Pan-European Trial for Adjuvant Treatment of Colon Cancer), to compare raltitrexed with the Mayo regimen of 5-FU and low-dose LV; (2) PETACC-2 (second Pan-European Trial), to compare the Mayo regimen with three regimens in which 5-FU is given by prolonged infusion. These trials will provide valuable international data to add to those from the USA and will assess the place of raltitrexed in the adjuvant treatment of Dukes' C colon cancer. They will also compare directly for the first time infusional and bolus 5-FU regimens in the adjuvant setting.

**Keywords:** colon adenocarcinoma; Mayo regimen; Pan-European Trial for Adjuvant Treatment of Colon Cancer; adjuvant treatment

Colorectal cancer is a major cause of mortality and morbidity in industrialized countries. Each year, more than 300 000 new cases are diagnosed and over 150 000 patients die of the disease in Europe and the USA (Estève et al, 1993; Boring et al, 1994). Of all patients who present with colorectal cancer, approximately three-quarters are potentially curable with radical resection of the primary tumour (Coperchini and Zalcberg, 1994–5). In spite of this, however, the prognosis associated with this malignancy remains poor, with at least 50% of patients dying of subsequent metastatic disease within 5 years (Coperchini and Zalcberg, 1994–5; Bleiberg, 1997). It is for this reason that adjuvant therapy (chemo-, radio- or immunotherapy) is added to surgery.

### ADJUVANT CHEMOTHERAPY IN COLON CANCER

Adjuvant treatment may be defined as treatment given in an attempt to eradicate clinically undetectable metastases, and to thus prevent local lymphatic spread and recurrence after potentially curative surgical resection of the primary tumour. The first adjuvant chemotherapy trials in colorectal cancer were carried out in the 1960s (van Triest et al, 1995). However, no significant survival benefit was shown for patients receiving adjuvant chemotherapy for many years (one of the reasons for early negative results may have been the low intensity of the treatments used in these studies); in addition, the majority of oncologists considered such treatment to be of no advantage for patients until the late 1980s when two large co-operative group trials showed significant benefits with adjuvant chemotherapy in patients with resected colon cancer (Wolmark et al, 1988; Laurie et al, 1989). In a trial with 401 patients with surgically treated Dukes' B2 or C disease, the North Central Cancer Treatment Group (NCCTG) showed a significant ($P = 0.003$) reduction in recurrence rate when the anthelmintic drug levamisole was given as adjuvant therapy in combination with 5-fluorouracil (5-FU). Patients were randomized to a control group (surgery alone) or to treatment with levamisole (150 mg/day for 3 days every 2 weeks for 1 year) or levamisole plus 5-FU (450 mg $m^{-2}$ bolus daily for 5 days followed by 450 mg $m^{-2}$ weekly, starting on day 28 and continued for 1 year). No statistically significant overall survival advantage was conferred by adjuvant treatment, but a retrospective subset analysis showed a significant survival advantage for levamisole plus 5-FU in patients with Dukes' C disease. Although the use of adjuvant chemotherapy remained controversial during this period, a breakthrough was finally made in 1990 when Moertel and colleagues (1990) published their pivotal findings in 318 evaluable patients with Dukes' B and 929 with Dukes' C disease.

In the Moertel study, 315 of the 929 patients with Dukes' C disease were randomized to a control group (surgery only with no further planned treatment), 310 to levamisole and 304 to levamisole plus 5-FU (same regimens as in the NCCTG study) (Laurie et al, 1989). After a median 3-year follow-up, therapy with levamisole plus 5-FU reduced the risk of recurrence of cancer by

*Correspondence to:* J Wils, Department of Internal Medicine/Oncology, Laurentius Hospital, 6043 CV Roermond, The Netherlands

'Tomudex' is a trademark, the property of Zeneca.

**Figure 1** (**A**) Three-year event-free survival and (**B**) overall survival after treatment with surgery only (control) or surgery and adjuvant chemotherapy with 5-fluorouracil plus leucovorin in patients with Dukes' B or C colon cancer in three randomized clinical trials. Median follow-up times were 37 and 40 months for adjuvant and control groups respectively (IMPACT, 1990)

an estimated 41% relative to control ($P < 0.0001$), whereas therapy with levamisole alone produced no significant effect. Rates of recurrence were reduced for all sites, but particularly for sites outside the abdominal cavity. The overall death rate was reduced with levamisole plus 5-FU by 33% ($P = 0.006$). No clear trends were apparent in patients with Dukes' B2 disease. These results were confirmed in a later report after a median follow-up period of 6.5 years (Moertel et al, 1995). These findings led the USA National Cancer Institute to convene a consensus conference at which the adoption of 5-FU plus levamisole as standard adjuvant therapy for Dukes' C colon cancer was recommended; ongoing trials that did not offer this treatment option were terminated (National Institutes of Health Consensus Development Conference Statement, 1990).

Further data to corroborate the above findings were presented in 1995 by the IMPACT (International Multicentre Pooled Analysis of Colon Cancer Trials) investigators (IMPACT, 1995). Results were pooled from three randomized multicentre co-operative group trials in 1526 patients with resected Dukes' B (56%) or C (44%) adenocarcinoma of the colon. Each trial used the same adjuvant treatment regimen of 5-FU 370–400 mg $m^{-2}$ plus leucovorin (LV) 200 mg $m^{-2}$ daily [Machover regimen (Machover et al, 1986)] for 5 days every 28 days for six cycles. Of the 1493 evaluable patients, 736 were in the adjuvant treatment group and 757 were in the control (surgery only) group. After median follow-up times of 40 and 37 months for the adjuvant chemotherapy and control groups, respectively, 5-FU plus LV was associated with significant reductions in mortality (22%, $P = 0.029$) and adverse events (35%, $P < 0.0001$), and increases in 3-year event-free survival (from 62% to 71%, hazard ratio 0.67, $P < 0.0001$) and overall survival (from 78% to 83%, hazard ratio 0.77, $P = 0.018$). Figure 1 illustrates survival for the different stages of disease and shows the much greater effect of adjuvant therapy in Dukes' C patients.

Improved survival with adjuvant chemotherapy was also shown by O'Connell and colleagues (1997) in a recent report of mature results from the NCCTG and the Mayo Clinic in the USA. In this study, 317 patients with high-risk TNM (tumour, node, metastases classification) stage II or III adenocarcinoma of the colon were randomized 3–4 weeks after surgery to adjuvant chemotherapy with six cycles of 5-FU 425 mg $m^{-2}$ plus low-dose LV (20 mg $m^{-2}$) (Mayo regimen) by rapid intravenous injection every day for 5 days every 4–5 weeks for six cycles or observation only. Of the evaluable patients, 62 of 151 control patients (41%) and 43 of 158 (27%) who had received chemotherapy had relapsed after a median 72 months ($P = 0.001$). In the control and adjuvant chemotherapy groups, mortality rates were 40% and 28% respectively ($P = 0.01$). The relative improvements seen in the patients treated with chemotherapy were sufficient after only 1 year for the investigators to discontinue the study prematurely on the grounds that it was no longer ethically justifiable to randomize patients to surgery alone.

Clinical studies have also been carried out to ascertain the relative efficacy of 5-FU modulated with levamisole, LV or both,

**Table 1** Adjuvant chemotherapy treatment regimens in NSABP trial C-04 in 2151 patients with Dukes' B or C colon cancer (Wolmark et al, 1996)

| Treatment arm | Regimen | Time on treatment |
|---|---|---|
| 5-FU + leucovorin (LV) | 5-FU 500 mg $m^{-2}$<br>LV 500 mg $m^{-2}$ weekly for 6 weeks | Six cycles |
| 5-FU + levamisole (LEV) | 5-FU 450 mg $m^{-2}$ daily for 5 days, then weekly after day 29 for 1 year<br>LEV 50 mg three times daily orally for 3 days every 2 weeks | 1 year |
| 5-FU + LV + LEV | 5-FU 500 mg $m^{-2}$ + LV 500 mg $m^{-2}$ weekly for 6 weeks<br>LEV 50 mg three times daily orally for 3 days every 2 weeks | 5-FU + LV, six cycles<br>LEV, 1 year |

© Cancer Research Campaign 1998

**Figure 2** Five-year disease-free survival (DFS) and overall survival in the NSABP C-04 study (Wolmark et al, 1996). Of 2151 patients who were randomized to treatment, 49% have contributed survival information over the mean 63.4-month follow-up period

**Figure 3** Survival rates after median follow-up of 4.2 years in 890 evaluable patients with high-risk TNM stage II or III resected colon cancer. The four adjuvant chemotherapy regimens were: (1) 5-fluorouracil 450 mg $m^{-2}$ daily for 5 days, then 450 mg $m^{-2}$ weekly from day 29; levamisole 50 mg orally three times daily for 3 days every 2 weeks for 6 months (5-FU/LEV 6 months); (2) as above for 12 months (5-FU/LEV 12 months); (3) 5-FU 370 mg $m^{-2}$ plus leucovorin 20 mg $m^{-2}$ daily for 5 days every 4–5 weeks; levamisole 50 mg orally three times daily for 3 days every 2 weeks for 6 months (5-FU/LV/LEV 6 months); (4) as above for 12 months (5-FU/LV/LEV 12 months)

given for periods of 6 or 12 months. The National Surgical Adjuvant Breast and Bowel Project (NSABP) group has recently reported on its protocol C-04, in which 2151 patients with resectable Dukes' B or C colon cancer were randomized to adjuvant chemotherapy with the three treatment options shown in Table 1 (Wolmark et al, 1996). Although there were no significant differences between the three arms in disease-free and overall survival, pairwise comparisons indicated an advantage for 5-FU modulated with LV (Figure 2).

Preliminary results are also available from a large USA study (INT-0089), started in 1988, in which patients with high-risk stage II or III (TNM) colon cancer were originally randomized to adjuvant therapy with 5-FU with high- or low-dose LV or to a control arm (surgery only) (Haller et al, 1996). Recruitment to the control arm was stopped in 1989 after reports of efficacy of adjuvant 5-FU plus levamisole (described earlier), and a 5-FU plus levamisole treatment arm was added to the trial protocol. A fourth arm was also added, in which patients received a combination of the standard levamisole regimen and 5-FU plus low-dose LV. The regimens are described in more detail in Table 2. A total of 3759 patients entered the trial, 80% of whom had stage III disease. After a median follow-up period of 3.8 years, three of the five planned treatment comparisons were mature, with no significant differences between treatments and no apparent additional benefit of levamisole added to 5-FU plus low-dose LV. A subsequent report from this trial stated that 6 months of adjuvant therapy with 5-FU plus LV was at least as effective as the standard 12-month regimen of 5-FU plus levamisole (Haller et al, 1997).

Results from a joint trial of the NCCTG and the National Cancer Institute of Canada (NCIC), in which 6- and 12-month regimens of 5-FU and LV were compared with 5-FU, LV and levamisole, showed that 12 months' chemotherapy offers no advantage over 6 months' treatment and that 5-FU plus levamisole for 6 months is inferior to 5-FU plus levamisole and LV for 6 months (Figure 3) (O'Connell et al, 1996).

### ADJUVANT CHEMOTHERAPY: THE CURRENT POSITION

These results strongly support the use of post-operative adjuvant chemotherapy, particularly in patients with Dukes' C colon cancer, although its use in Dukes' B disease remains controversial. Available data indicate 5-FU with LV, given for 6 months, to be at least as effective as 5-FU plus levamisole for 12 months. Thus, the

**Table 2** Adjuvant chemotherapy treatment regimens in INT-0089 in 3759 patients with TNM stage II or III colon cancer (Haller et al, 1996)

| Treatment arm | Regimen | Time on treatment (months) |
|---|---|---|
| 5-FU + low-dose (LD) leucovorin (LV) | 5-FU 425 mg $m^{-2}$ daily for 5 days<br>LV 20 mg $m^{-2}$ daily for 5 days; weeks 1, 5, 9, 14, 19 and 24 | 6 |
| 5-FU + high-dose (HD) LV | 5-FU 500 mg $m^{-2}$ weekly for 6 weeks<br>LV 500 mg $m^{-2}$ weekly for 6 weeks; every 2 months for four cycles | 8 |
| 5-FU + levamisole (LEV) | 5-FU 450 mg $m^{-2}$ daily for 5 days, then every week for 12 months beginning at week 5<br>LEV 50 mg three times daily orally for 3 days every 2 weeks | 12 |
| 5-FU + LDLV + LEV | 5-FU 425 mg $m^{-2}$ daily for 5 days<br>LV 20 mg $m^{-2}$ daily for 5 days<br>LEV 50 mg three times daily orally for 3 days every 2 weeks | 12 |

*© Cancer Research Campaign 1998*

**Table 3** Groups participating in the Pan-European adjuvant chemotherapy studies

| Country | Group | Initials/acronym | Chief investigators |
|---|---|---|---|
| Canada | Canadian Group for Colorectal Cancer | CGCRC | M Vincent |
| Egypt | Egyptian Cancer Society | ECS | M El-Sarafy, H Khaled, S Omar |
| Europe | European Organization for Research and Treatment of Cancer – Gastrointestinal Tract Cancer Co-operative Group | EORTC – GITCCG | J Wils, D Nitti, B Paillot, R Sylvester |
| France | Fondation Française de Cancérologie Digestive | FFCD | L Bedenne, JF Seitz |
| Germany | Arbeitsgemeinschaft Internistische Onkologie/Chirurgische Arbeitsgemeinschaft Onkologie | AIO/CAO | K Höffken, H Wilke, C-H Köhne, H-J Meyer, K Lorenz |
| Italy | Gruppo Interdisciplinare Valutazione Interventi in Oncologia; Gruppo Italiano Studio Carcinomi Apparato Digerenti | GIVIO; GISCAD | R Labianca, V Torri |
| | Intergruppo Nazionale Terapia Adjuvante Carcinoma Colon | INTACC (GOIRC, GOPTAD, IOR, IST Genova) | A Sobrero, F Di Costanzo, P Bruzzi, L Dogliotti, A Falcone, L Frassinetti, R Rosso |
| | Gruppo Oncologico-Chirurgico Cooperativo Italiano | GOCCI | F Tonelli, T Mazzei, E Mini |
| Portugal | Grupo Cooperativo Cancro Digestivo; Associao Portuguesa Investigaca Oncologio | GCCD; APIO | J Guimaraes Dos Santos, E Sanches |
| Spain | Grupo Español Tratamiento Tumores Digestivos | TTD | A Carrato, E Diaz-Rubio, E Aranda |
| UK | Clinical Trials Unit | QUASAR | D Kerr, C McArdle, R Gray |

Mayo regimen of 5-FU 425 mg m$^{-2}$ (370 mg m$^{-2}$ in some centres) plus LV 20 mg m$^{-2}$ for 5 days every 4–5 weeks (Poon et al, 1989) is currently considered to be the preferred adjuvant treatment in patients with resected Dukes' C colon cancer. However, regimens based on 5-FU are complicated to administer and are associated with troublesome toxicity, particularly mucositis and leucopenia (Petrelli et al, 1987, 1989), which necessitates dose reduction and/or delay in treatment (Advanced Colorectal Cancer Meta-analysis Project, 1992). Indeed, in one of the above studies (Wolmark et al, 1996), toxicity of WHO grade 3 severity or greater was reported in 36% of patients who received 5-FU plus LV, 38% of those who received 5-FU, LV and levamisole and 28% of those who received 5-FU plus levamisole. Thus, there is interest in the use of agents in adjuvant therapy that are as effective as modulated 5-FU but that have more favourable tolerability profiles and less complex administration schedules.

Raltitrexed, a direct and specific inhibitor of thymidylate synthase (TS), is an interesting new candidate for the adjuvant therapy of surgically managed cancer of the colon. Raltitrexed has already been shown in phase II and III studies to be of similar efficacy to 5-FU modulated with LV in the management of advanced colorectal cancer (Kerr, 1997; Blackledge, 1998). Palliative benefits were reported with both drugs, and raltitrexed was associated with tolerability advantages compared with 5-FU, especially in early treatment cycles, which reflected significant quality-of-life benefits for raltitrexed in cycle 1.

Diarrhoea, mucositis, leucopenia and alopecia were markedly less frequent and less severe with raltitrexed than with 5-FU + LV in all phase III studies. Raltitrexed was associated with elevated hepatic transaminase levels, but these were of no clinical consequence and declined with continued treatment. The reduced frequency of clinically significant adverse effects with raltitrexed was reflected by significantly fewer toxicity-related dosage reductions in early treatment cycles in patients treated with raltitrexed (Zalcberg, 1997). The convenient administration schedule for this drug (one 15-min infusion every 3 weeks) is also of interest, as it reduces the frequency of hospital attendance by patients with consequent beneficial effects on quality of life and on healthcare resource consumption. These benefits of therapy with raltitrexed may prove particularly advantageous in the adjuvant setting, in which patients may be asymptomatic before chemotherapy.

**Figure 4** Regimens for high-dose infusional 5-fluorouracil in PETACC-2

### NEW EUROPEAN CLINICAL STUDIES OF ADJUVANT RALTITREXED AND 5-FU IN DUKES' C COLON CANCER

Large studies of the efficacy of adjuvant chemotherapy in resected colon cancer have, to date, been carried out by investigators in the USA. Non-USA data in this area of study are scarce. Two

© Cancer Research Campaign 1998

international studies have been proposed to determine the efficacy of raltitrexed and various infusional 5-FU regimens relative to currently accepted adjuvant treatment. In order to recruit the large numbers of patients needed, these studies will involve the collaboration of international co-operative groups with expertise in the study and treatment of colorectal cancer (Table 3).

The first Pan-European Trial for Adjuvant Treatment of Colon Cancer (PETACC-1), which will compare raltitrexed with the Mayo (low-dose LV) regimen as post-surgical adjuvant treatment in patients with Dukes' C colon cancer who have undergone curative radical resection within the 42 days preceding enrolment, was initiated in February 1998. Primary objectives are to determine recurrence-free and overall survival rates; the secondary objective will be to compare the toxicities of the two regimens. PETACC-1 is a randomized, multicentre, international, intergroup phase III trial that will be conducted in approximately 400 centres. The recruitment target is around 3000 patients with histologically confirmed Dukes' C adenocarcinoma of the colon. Each patient will be required to have received no prior chemotherapy and to have a WHO performance status of 0 to 1, and will be randomized to raltitrexed 3 mg $m^{-2}$ as a 15-min intravenous infusion once every 3 weeks for eight cycles or bolus 5-FU 425 or 370 mg $m^{-2}$ with LV 20 mg $m^{-2}$ for 5 days every 4 weeks for six cycles. Treatment will last for 24 weeks.

Patients will be assessed at least once every 6 months for the first 3 years after randomization of the last patient and at least once a year thereafter until death. To maintain the statistical power of the study, recurrence-free and overall survival will not be finally analysed until a total of 703 events/deaths have been reported. It is anticipated that analyses will be carried out at 1.5 (for recurrence-free survival) and 3 (for survival) years and that results will be available in the year 2002.

A second study, PETACC-2, will be carried out at the same time to compare the Mayo regimen (5-FU 370 or 425 mg $m^{-2}$ plus LV 20 mg $m^{-2}$ for 5 days every 4 weeks for six cycles) with three high-dose infusional 5-FU regimens. Interest in such a comparison stems from observations indicating that 5-FU acts in different ways depending on whether it is administered over a short period as an intravenous bolus injection or rapid infusion (e.g. up to 15 min) or as a prolonged infusion (Sobrero et al, 1997). Three infusion regimens are being assessed in the infusion arm of the trial (Figure 4); this itself is noteworthy, because these regimens have never been directly compared, but the assumption is that no major differences between the three regimens exist.

### CONCLUSIONS

This is an exciting and interesting time for all those involved in the treatment of resected adenocarcinoma of the colon. Now that the benefit of adjuvant chemotherapy to patients with Dukes' C disease has been clarified in USA centres, it is time to move to the next stage of research to assess the benefit of alternatives to the Mayo regimen of 5-FU with low-dose LV. These alternatives include raltitrexed and various infusional 5-FU regimens. To match the power of the USA studies, it is necessary to organize collaborative efforts with the global involvement of experienced teams. It is anticipated that PETACC-1 and PETACC-2 will further enhance our understanding of and ability to treat resectable adenocarcinoma of the colon, and will provide valuable insight into new treatment regimens that may have advantages over existing therapies.

### REFERENCES

Advanced Colorectal Cancer Meta-Analysis Project (1992) Modulation of fluorouracil by leucovorin in patients with advanced colorectal cancer: evidence in terms of response rate. *J Clin Oncol* **10**: 896–903

Blackledge G (1998) New developments in cancer treatment with the novel thymidylate synthase inhibitor raltitrexed ('Tomudex'). *Br J Cancer* (this suppl.)

Bleiberg H (1997) Colorectal cancer: is there an alternative to 5-FU? *Eur J Cancer* **33**: 536–541

Boring CC, Squires TS, Tong T and Montgomery S (1994) Cancer Statistics, 1994. *CA Cancer J Clin* **44**: 7–26

Coperchini ML and Zalcberg J (1994–95) Overview of the current treatment of colorectal cancer. *Diagn Oncol* **4**: 130–139

Estève J, Kricker A, Ferlay J and Parkin DM (1993) In *Facts and Figures of Cancer in the European Community*. International Agency for Research on Cancer: Lyon

Haller DG, Catalano PJ, Macdonald JS and Mayer RJ (1996) Fluorouracil (FU), leucovorin (LV) and levamisole (LEV) adjuvant therapy for colon cancer: preliminary results of INT-0089 (abstract 486). *Proc Am Soc Clin Oncol* **15**: 211a

Haller DG, Catalano PJ, Macdonald JS and Mayer RJ (1997) Fluorouracil (FU), leucovorin (LV) and levamisole (LEV) adjuvant therapy for colon cancer: four-year results of INT-0089 (abstract 940). *Proc Am Soc Clin Oncol* **16**: 265a

International Multicentre Pooled Analysis of Colon Cancer Trials Investigators (1995) Efficacy of adjuvant fluorouracil and folinic acid in colon cancer. *Lancet* **345**: 939–944

Kerr DJ (1997) Clinical efficacy of 'Tomudex' (raltitrexed) in advanced colorectal cancer. *Anticancer Drugs* **8** (suppl. 2): S11–S15

Laurie JA, Moertel CG, Fleming TR, Wieand HS, Leigh JE, Rubin J, McCormack GW, Gersmer JB, Krook JE, Malliard J, Twito DI, Morton RF, Tschetter LK and Barlow JF (1989) Surgical adjuvant therapy of large-bowel carcinoma: an evaluation of levamisole and the combination of levamisole and fluorouracil: the North Central Cancer Treatment Group and the Mayo Clinic. *J Clin Oncol* **7**: 1447–1456

Machover D, Goldsmith E, Chollet P, Metzger G, Zittoun J, Marquet J, Vandenbulcke J-M, Misset J-L, Schwarzenberg L, Fourtillan JB, Gaget H and Mathé G (1986) Treatment of advanced colorectal and gastric adenocarcinoma with 5-fluorouracil and high dose folinic acid. *J Clin Oncol* **4**: 685–696

Moertel CG, Fleming TR, Macdonald JS, Haller DG, Laurie JA, Goodman PJ, Ungerleider JS, Emerson WA, Tormey DC, Glick JH, Veeder MH and Mailliard JA (1990) Levamisole and fluorouracil for adjuvant therapy of resected colon carcinoma. *N Engl J Med* **8**: 352–358

Moertel CG, Fleming TR, Macdonald JS, Haller DG, Laurie JA, Tangen CM, Ungerleider JS, Emerson WA, Tormey DC, Glick JH, Veeder MH and Mailliard JA (1995) Fluorouracil plus levamisole as effective adjuvant therapy after resection of stage III colon carcinoma: a final report. *Ann Intern Med* **122**: 321–326

National Institutes of Health Consensus Development Conference Statement, April 16–18 (1990) *Adjuvant Therapy for Patients with Colon Cancer and Rectum Cancer*. Office of Medical Applications of Research. National Institutes of Health: Bethesda, MD

O'Connell M, Laurie JA, Shepherd L, Kahn MJ, Pazdur R, Fitzgibbons RJ, Erlichman C and Wieand HS (1996) A prospective evaluation of chemotherapy duration and regimen as surgical adjuvant treatment for high-risk colon cancer: a collaborative trial of the North Central Cancer Treatment Group and the National Cancer Institute of Canada trials (abstract 478). *Proc Am Soc Clin Oncol* **15**: 209a

O'Connell MJ, Mailliard JA, Kahn MJ, Macdonald JS, Haller DG, Mayer RJ and Wieand HS (1997) Controlled trial of flurorouracil and low-dose leucovorin given for 6 months as postoperative adjuvant therapy for colon cancer. *J Clin Oncol* **15**: 246–250

Petrelli N, Herrera L, Rustum Y, Burke P, Creaven P, Stulc J, Emrich LJ and Mittelman A (1987) A prospective randomized clinical trial of 5-fluorouracil versus 5-fluorouracil and high dose leucovorin versus fluorouracil and methotrexate in previously untreated patients with advanced colorectal cancer. *J Clin Oncol* **5**: 1559–1565

Petrelli N, Douglas Jr HO, Herrera L, Russell D, Stablein DM, Bruckner HW, Mayer RJ, Schinella R, Green MD and Muggia FM (1989) The modulation of fluorouracil with leucovorin in metastatic colorectal carcinoma: a prospective randomized phase III trial. *J Clin Oncol* **7**: 1419–1426

Poon MA, O'Connell MJ, Wieand HS, Cullinan SA, Everson LK, Krook JE, Mailliard JA, Laurie JA, Tschetter LK and Wiesenfeld M (1989) Biochemical modulation of fluorouracil: evidence of significant improvements in survival and quality of life in patients with advanced colorectal carcinoma. *J Clin Oncol* **7**: 1407–1418

Sobrero AF, Aschele C and Bertino JR (1997) Fluorouracil in colorectal cancer – a tale of two drugs: implications for biochemical modulation. *J Clin Oncol* **15**: 368–381

van Triest B, van Groeningen CJ and Pinedo HM (1995) Current chemotherapeutic possibilities in the treatment of colorectal cancer. *Eur J Cancer* **7/8**: 1193–1197

Wolmark N, Fisher B, Rockette H, Redmond C, Wickerham DL, Fisher ER, Jones J, Glass A, Lerner H and Lawrence W (1988) Postoperative adjuvant chemotherapy or BCG for colon cancer: results from NSABP protocol C-01. *J Natl Cancer Inst* **80**: 30–36

Wolmark N, Rockette H, Mamounas EP, Jones J, Petrelli N, Atkins J, Dimitrov N, Pugh R, Wickerham DL, Wieand HS and Fisher B (1996) The relative efficacy of 5-FU + leucovorin (FU-LV), 5-FU + levamisole (FU-LEV), and 5-FU + leucovorin + levamisole (FU-LV-LEV) in patients with Dukes' B and C carcinoma of the colon: first report of NSABP C-04 (abstract 460). *Proc Am Soc Clin Oncol* **15**: 205a

Zalcberg JR (1997) Overview of the tolerability of 'Tomudex' (raltitrexed): collective clinical experience in advanced colorectal cancer. *Anti-Cancer Drugs* **8**(suppl.2): S17–S22

*© Cancer Research Campaign 1998*